# Oral cancer knowledge, attitudes, and practices among dentists in Kabul, Afghanistan

**DOI:** 10.1186/s12903-026-08258-x

**Published:** 2026-04-07

**Authors:** Ahmad Khalid Aalemi, Ahmad Shekib Sobat, Fariha Kamal, Noor Ahmad Shah Noor, Alison K. Wright

**Affiliations:** 1 Present Address: Department of Public Health Sciences, School of Medicine, University of California Davis, Sacramento, CA USA; 2 Department of Oral Surgery, Kabul University of Medical Sciences, Kabul, 1001 Afghanistan; 3 Department of Oral Medicine, Kabul University of Medical Sciences, Kabul, 1001 Afghanistan; 4 Division of Pharmacy and Optometry, School of Health Sciences, Faculty of Biology, Medicine and Health, University of Manchester, Manchester, UK

**Keywords:** Oral cancer, Knowledge, Dentists, Kabul

## Abstract

**Background:**

Oral cancer is a major global health concern, with low survival due to late diagnosis and insufficient knowledge among dental professionals. This study assessed oral cancer-related knowledge, attitudes, and practices among dentists in Kabul, Afghanistan.

**Methods:**

A cross-sectional study was conducted among 212 licensed dentists practicing at stomatology hospitals or private dental clinics in Kabul between January and September 2025. Data was collected using a validated, self-administered questionnaire including socio-demographic information. Responses were anonymous and analyzed with descriptive statistics, chi-square tests and multivariate logistic regression; *P* < 0.05 was considered statistically significant.

**Results:**

Participants were predominantly male (69.3%) and aged 23–29 years (65.1%). Over half had less than five years of professional dental experience (53.3%), and most practiced general dentistry (64.2%) in private clinics (66.2%). Continuing Medical Education (CME) participation was limited, with 83% reporting no prior attendance. Clinical knowledge of oral cancer was moderate (mean 6.28 ± 2.35), with 65.6% scoring low and 34.4% medium/high. Knowledge of oral cancer risk factors was higher (mean 9.13 ± 1.66), with 67.5% demonstrating medium/high knowledge. Major behavioral risk factors, including tobacco, alcohol, and Naswar use, were widely recognized, whereas awareness of age-, diet-, and viral-related risks were more limited Most dentists referred suspicious lesions to specialists (43.4%), but few had performed biopsies or referrals in the past year. Assessment of patient risk factors was inconsistent, with more focus on tobacco use than alcohol consumption or cancer history. Clinical knowledge of oral cancer was significantly associated with the dentist’s experience and place of practice, with higher knowledge among dentists with > 10 years of experience, and those working in government hospitals. Risk factor knowledge showed no significant associations.

**Conclusions:**

Dentists practicing in Kabul demonstrated moderate oral cancer knowledge, relatively low levels of engagement in preventive practices, and infrequent CME participation, highlighting areas where targeted education may help support early detection and management of oral cancer.

## Background

Oral cancer is among the most prevalent malignancies in the head and neck region, with global epidemiological trends showing a substantial and continuing increase in disease burden, posing a growing public health challenge [1]. Annually, 389,846 new cases are reported worldwide, accounting for 2% of all new cancer cases [2]. Oral cancer poses a major health issue, particularly in South and Southeast Asia where incidence and mortality rates are among the highest globally (South-East Asia incidence: 8.1 per 100,000 person-years [2]. Epidemiological studies indicate that smoking and alcohol consumption are the primary risk factors, contributing substantially to the high incidence, especially among younger individuals. Additionally, the use of smokeless tobacco products, such as paan, betel quid, and Naswar, also pose a significant risk for oral and pharyngeal cancers [3].

Despite advancements in the diagnosis and treatment of oral cancer, the overall 5-year survival rate for patients remains between 40% and 63% [4, 5]. A lack of awareness and delays in referral contribute to the majority of oral cancer cases being diagnosed at an advanced stage [6]. Consequently, despite undergoing extensive surgery, radiotherapy, chemotherapy, and facing significant financial burdens, patients often have a limited prognosis. However, early detection of oral cancer may lead to improved treatment success rates, higher survival rates, and more favorable functional outcomes [7].

Early diagnosis of oral cancer is theoretically feasible, given the visibility of the mouth. However, insufficient knowledge and low awareness of oral cancer often lead to late diagnosis. Approximately half of oral cancer cases are identified at a late stage, underscoring the critical need for increased awareness and knowledge on this issue [8].

Healthcare professionals, particularly dental practitioners, bear significant responsibilities in the prevention and early detection of oral cancer. Motivating patients to abandon harmful habits that can lead to oral cancer is as crucial as treating the disease itself. Numerous studies have documented the awareness and understanding of oral cancer among dentists in various countries [9–20]. There is paucity of similar studies conducted among dentist of Afghanistan.

The World Health Organization emphasizes that decreasing the incidence of oral cancer necessitates a comprehensive strategy encompassing health education, literacy, risk factor reduction, and early diagnosis [21]. Within this framework, this study aims to evaluate oral cancer awareness and provide a detailed assessment of current knowledge about oral cancer among dentists in Kabul Afghanistan.

## Methods

A cross-sectional study was conducted among 212 dentists practicing in Kabul City between January and September 2025. The sample size was calculated using a finite population correction formula with a population of 400 dentists, 95% confidence level, 5% margin of error, and *P* = 50%. The required sample size was 196, and after adding 10% for potential non-response, the final sample size was 216 participants. In Kabul city, several areas have a high concentration of dental clinics, and dentists from these areas were selected using convenience sampling. Dentists holding a valid license to practice and providing informed consent were included; those who declined participation were excluded. Questionnaires were distributed in person at dental workplaces with a trained research assistant available to provide clarification when needed. Responses were anonymous and self-administered.

Data were collected using a structured, self-administered questionnaire adapted from international studies assessing oral cancer–related knowledge, attitudes, and practices [10, 11, 14–16, 18, 22–24]. The instrument comprised four sections: (1) socio-demographic and professional characteristics, (2) knowledge of clinical presentation, (3) knowledge of risk factors, and (4) attitudes and clinical practices toward oral cancer examination. The questionnaire underwent face validity review by a panel of 3 experts, including dentists and public health specialists, and minor revisions were made to improve clarity and relevance. Pilot testing was conducted among10 of dentists, and a Farsi translation was provided alongside the original version. There were 14 items to assess knowledge of clinical presentation, and 16 items to assess knowledge of risk factors. Each correct response received one point, and knowledge levels were categorized using an established scoring scale: clinical presentation (0–9 low, 10–11 medium, 12–14 high) and risk factors (0–8 low, 9–10 medium, 11–16 high), with medium and high scores considered satisfactory [24]. The attitude section comprised 11 items designed to assess dentists’ perceptions of their role in oral cancer prevention. Responses were recorded on a 5-point Likert scale (1 = strongly agree, 2 = agree, 3 = disagree, 4 = strongly disagree, 5 = don’t know). The clinical practice section included 13 items addressing dentists’ procedures for oral cavity examination, the factors they consider when reviewing a patient’s medical history, and the types of educational materials available for patient use.

Data entry, cleaning, and coding were performed using IBM SPSS Statistics Version 31. Descriptive statistics were used to summarize demographic characteristics and questionnaire responses. The Pearson chi-square test was used to assess the association between knowledge levels and dentists’ characteristics. Multivariate logistic regression was employed to identify potential predictors of satisfactory versus unsatisfactory knowledge of OC. A *p*-value of < 0.05 was considered statistically significant.

Ethical approval was obtained from the Institutional Review Board of Kabul University of Medical Sciences [12/18; 17/11/2024]. Written informed consent was obtained prior to participation, and all responses were anonymized. Ethical procedures adhered to the principles outlined in the Declaration of Helsinki.

## Results

Table 1 presents the socio-demographic characteristics of the participating dentists. The majority were male (69.3%) and relatively young, with 65.1% aged 23–29 years. Over half of the participants (53.3%) had less than five years of professional experience, and most practiced in general dentistry (64.2%). Two-thirds worked in private dental clinics, while the remainder were employed in government hospitals or private hospitals. Attendance at continuing medical education (CME) was infrequent, with 83.0% of dentists reporting they had never attended CME activities (Table 1).

**Table 1:** Socio-demographic characteristics of the participants

Characteristics	Total participants (*N* = 212)	
	*N*	%
Sex		
Male	147	69.3
Female	65	30.7
Age, years		
23–29	138	65.1
30–39	54	25.5
40–49	16	7.5
50–59	4	1.9
Years of professional experience		
Less than 5 years	113	53.3
5–10 years	73	34.4
11–15 years	20	9.4
More than 15 years	6	2.8
Scope of practice		
General dental practice	136	64.2
Specialty dental practice	76	35.8
Place of practice		
Government hospital	47	22.2
Private hospital	24	11.3
Private dental clinic	141	66.5
Last attended CME		
Within the past 2 years	19	9.0
During the past 2–5 years	10	4.7
More than 5 years ago	7	3.3
Never	176	83.0

### Clinical presentation awareness

Table 2 summarizes dentists’ knowledge of oral cancer clinical presentations. The mean score for dentists’ knowledge of clinical presentations was 6.28 ± 2.35, ranging from 1 to 11. The majority of dentists had low scores (*n* = 139, 65.6%), and only 34.4% (*n* = 73) achieved medium to high scores. Most of them correctly identified oral and tongue examinations (81.6% and 83.5%, respectively) and recognized squamous cell carcinoma as the most common type of oral cancer (74.1%). However, knowledge about specific risk factors, early signs, and sites of oral cancer was limited. Only 38.2% identified the tongue as the most common site, 12.3% correctly noted the ventral-lateral border of the tongue as a frequent site, and 4.2% recognized that early oral cancer can be asymptomatic. Approximately two-thirds (66.5%) acknowledged that oral cancer is often diagnosed at an advanced stage. Recognition of premalignant lesions was also variable, with 41.5% identifying erythroplakia and leukoplakia as associated with oral cancer, but only 21.7% correctly identifying erythroplakia as the more serious premalignant condition. Knowledge about other clinical features, including familial clustering, age of diagnosis, lymph node involvement, and lip cancer related to sun exposure, ranged between 25% and 48%.

**Table 2:** Distribution of dentists based on their ability to correctly identify clinical presentations of oral cancer

	*N*	%
Correctly identify oral examination	173	81.6
Correctly identify tongue examination	177	83.5
The tongue is the first most common site of oral cancer	81	38.2
Squamous cell carcinoma is the most common type of oral cancer	157	74.1
Familial clustering is least likely to be associated with oral cancer	100	47.2
Early signs of oral cancer is often asymptomatic	9	4.2
Majority of oral cancers are diagnosed in people 60 years or older	54	25.5
Hard, painless, mobile or fixed lymph node is most characteristic of oral cancer metastasis	101	47.6
Ventral - lateral border of the tongue is the most likely site to develop oral cancer	26	12.3
Oral cancer is most often diagnosed in advanced stages	141	66.5
Lip cancer is related to sun exposure	101	47.6
Early oral cancer lesions appear as small, painless, red areas	78	36.8
Erythroplakia and leukoplakia are associated with oral cancer	88	41.5
Erythroplakia is a more serious premalignant condition than leukoplakia.	46	21.7

### Risk factor awareness

**Fig. 1:**
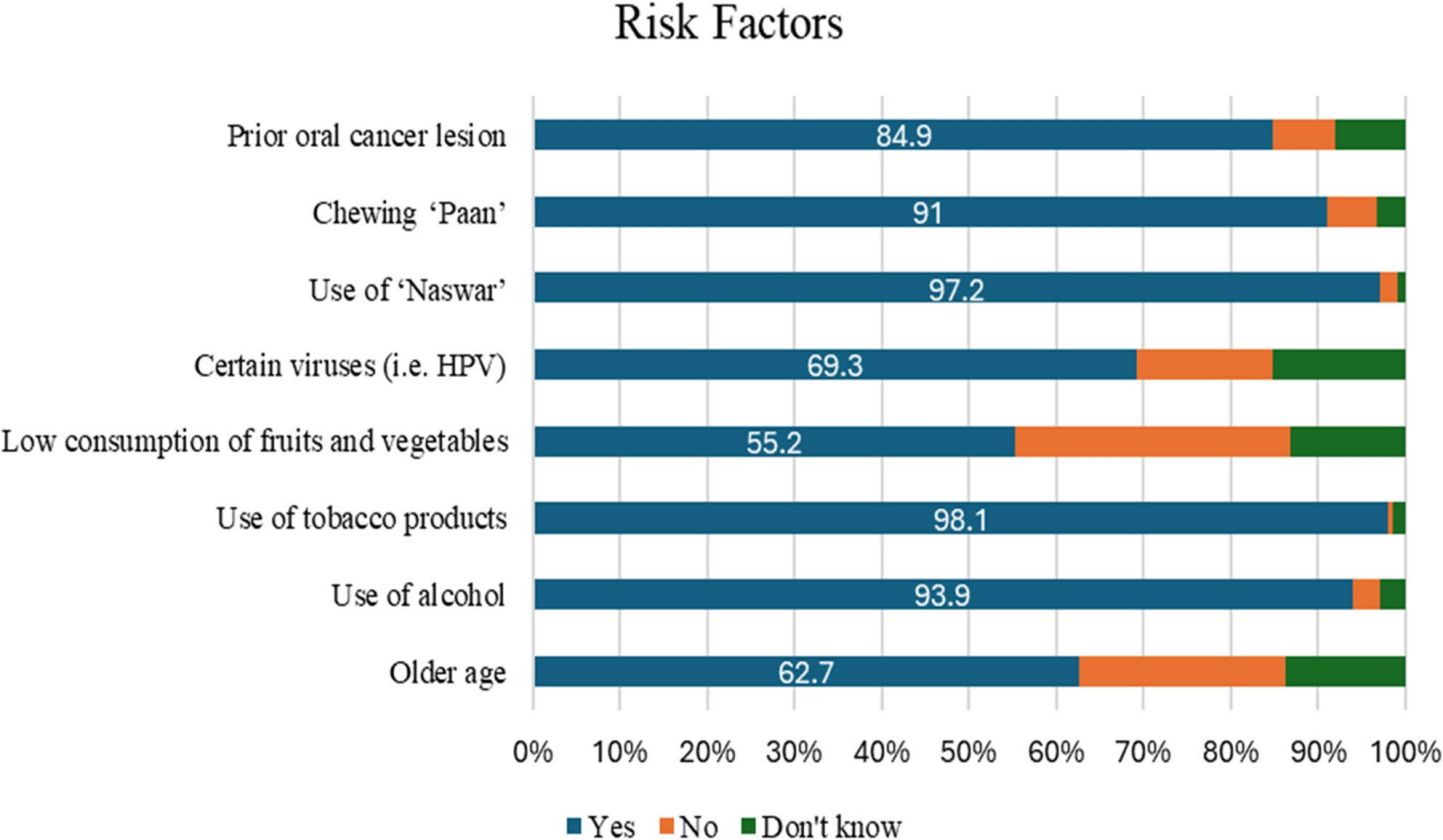
Distribution of dentists that correctly identified the listed oral cancer risk factors

**Fig. 2:**
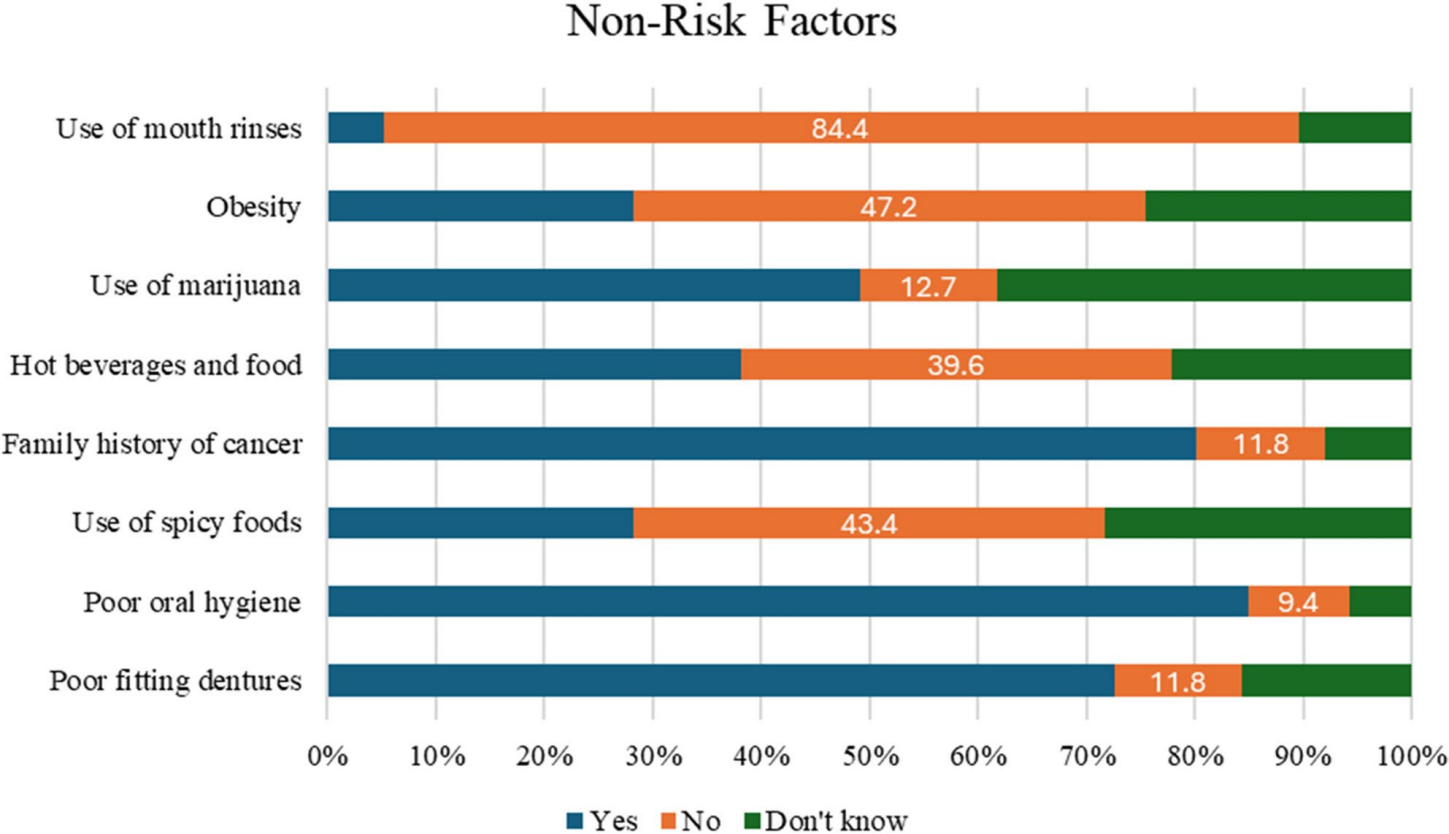
Distribution of dentists that correctly identified the listed oral cancer non-risk factors

Knowledge levels of risk factors and non-risk factors (misconceptions) of oral cancer are shown in Figs. 1 and 2, respectively. The mean knowledge score for oral cancer risk factors was 9.13 ± 1.66, ranging from 3 to 14. Most participants demonstrated medium to high knowledge (*n* = 143, 67.5%), while 32.5% (*n* = 69) had low knowledge. The dentists demonstrated strong awareness of major behavioral risk factors but had relatively lower recognition of age- and diet-related risks (Fig. 1). Most of the dentists correctly identified the use of tobacco products (98.1%), ‘Naswar’ (97.2%), alcohol consumption (93.9%), chewing ‘Paan’ (91.0%), and prior oral cancer lesions (84.9%) as significant risk factors. Recognition of viral infections such as HPV was moderate (69.3%), while older age (62.7%) and low consumption of fruits and vegetables (55.2%) were less frequently acknowledged as risk factors.

Generally, the dentists demonstrated good awareness of established non-risk factors but some misconceptions regarding diet, lifestyle, and genetic influences were observed (Fig. 2). Most correctly identified poor oral hygiene (84.9%), use of mouth rinses (84.4%), family history of cancer (80.2%), and poor-fitting dentures (72.6%) as non-risk factors. Recognition was lower for obesity (47.2%), spicy foods (43.4%), hot beverages (39.6%), and marijuana use (12.7%).

### Oral cancer examination attitudes and practices

**Fig. 3:**
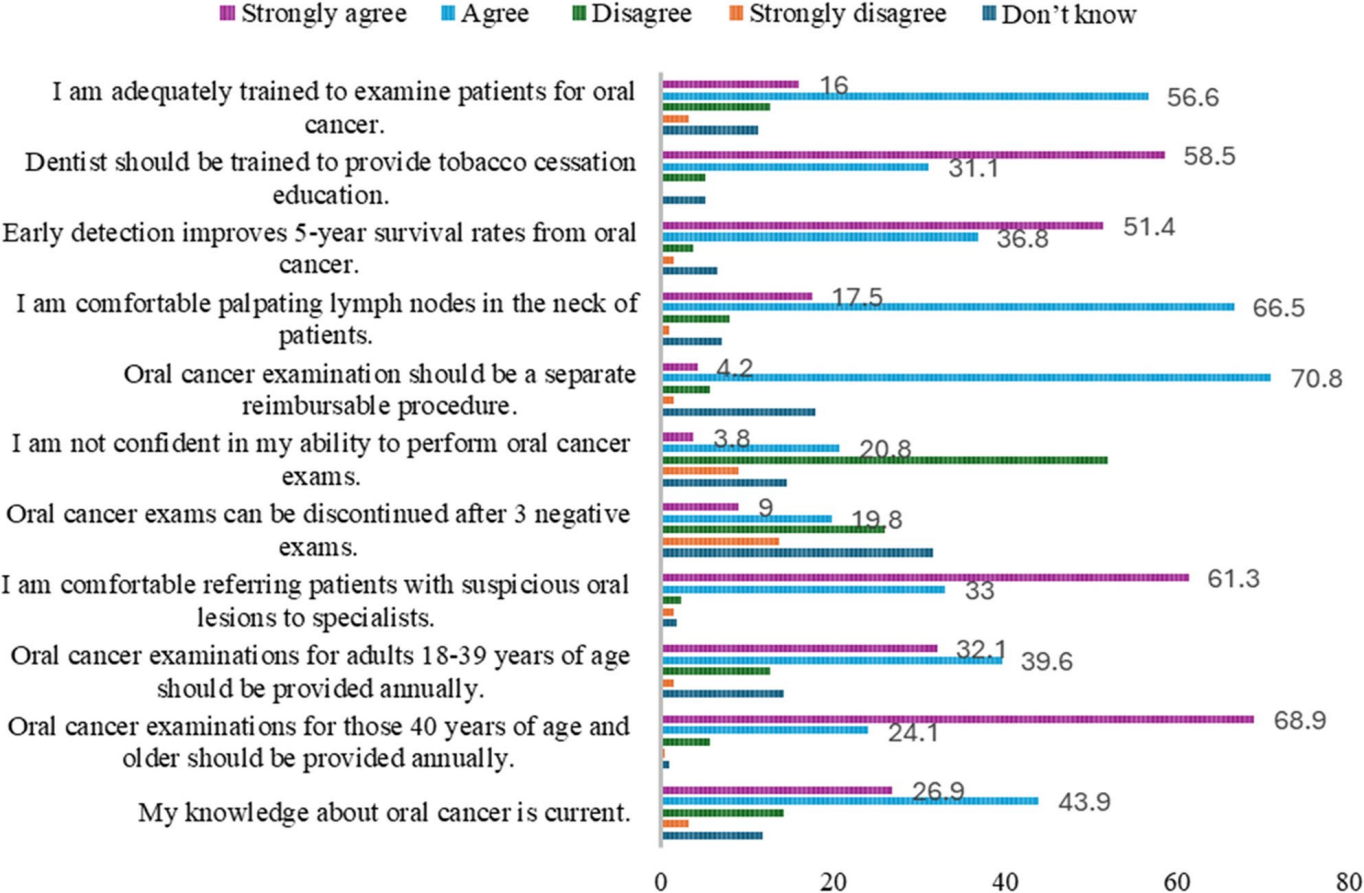
Attitude of dentists toward oral cancer examination

Most participants agreed that oral cancer examinations for adults aged 40 years and older should be provided annually (68.9% strongly agree, 24.1% agree) and were comfortable referring patients with suspicious lesions to specialists (61.3% strongly agree, 33.0% agree). The majority also acknowledged the importance of early detection for improving 5-year survival rates (51.4% strongly agree, 36.8% agree) and supported training dentists to provide tobacco cessation education to patients (58.5% strongly agree, 31.1% agree) (Fig. 3).

However, confidence in performing oral cancer exams varied: over 72% of participants felt they were adequately trained, 66.5% were comfortable palpating lymph nodes in patients’ necks, and 51.9% disagreed or strongly disagreed about lacking confidence. Most dentists disagreed with discontinuing exams of suspicious lesions after three negative results (25.9% disagree, 13.7% strongly disagree). Additionally, 70.8% agreed that oral cancer examination should be a separate reimbursable procedure.

Table 3 summarizes dentists’ clinical practices related to oral cancer detection and patient management. When a suspicious lesion was detected, most dentists referred patients to specialists (43.4%), while others performed an incisional biopsy (33.0%) or a brush biopsy (20.3%). During the previous 12 months, most dentists reported neither performing a biopsy (76.9%) nor making a referral (72.2%) for suspicious lesions. Perceptions of responsibility for early oral cancer detection were divided, with 46.7% attributing this role to general dentists and 49.1% to oral and maxillofacial surgeons.

**Table 3:** Distribution of dentists according to their clinical practice regarding oral cancer

	*N*	%
When you detect a lesion, do you:		
Perform Toluidine Blue Staining	6	2.8
Perform Brush Biopsy	43	20.3
Perform Incisional Biopsy	70	33.0
Use ViziLite	1	0.5
Refer	92	43.4
In the past 12 months, about how many patients did you biopsy for suspicious oral lesions		
Zero	163	76.9
1–5	39	18.4
More than 5	10	4.7
In the past 12 months, about how many patients did you refer for diagnosis of suspicious oral lesions		
Zero	153	72.2
1–5	46	21.7
More than 5	13	6.1
In your opinion, who should have the primary role in detecting early signs and symptoms of oral cancer		
General Dentist	99	46.7
Oral and maxillofacial surgeon	104	49.1
Dermatologist	2	0.9
Ear, nose and throat specialist	7	3.3
What kind of oral cancer patient education materials are available in your practice		
None	146	68.9
Brochures/Pamphlets	51	24.1
Video	15	7.1
When taking a medical history, do you assess patient’s past alcohol use		
Yes	122	57.5
No	90	42.5
When taking a medical history, do you assess patient’s present alcohol use		
Yes	127	59.9
No	85	40.1
When taking a medical history, do you assess type & amount of alcohol used		
Yes	82	38.7
No	130	61.3
When taking a medical history, do you assess patient’s previous tobacco use		
Yes	179	84.4
No	33	15.6
When taking a medical history, do you assess patient’s present tobacco use		
Yes	186	87.7
No	26	12.3
When taking a medical history, do you assess type & amount of tobacco		
Yes	117	55.3
No	95	44.8
When taking a medical history, do you assess patient’s history of cancer		
Yes	126	59.4
No	86	40.6
When taking a medical history, do you assess family history of cancer		
Yes	128	60.4
No	84	39.6

Assessment of patient risk factors during medical history-taking varied. Most dentists evaluated past and current tobacco use (84.4% and 87.7%, respectively), whereas fewer assessed the type and amount of alcohol consumed (38.7%) or tobacco used (55.3%). Assessment of cancer history was moderate, with 59.4% evaluating personal history and 60.4% assessing family history. Patient education resources were limited, with 68.9% of dentists reporting having no educational materials available.

### Association between dentists’ characteristics and oral cancer knowledge

Table 4 summarizes the distribution of dentists’ clinical and risk factor knowledge of oral cancer according to their demographic and professional characteristics. Clinical knowledge differed significantly by years of practical experience (*P* = 0.015), place of practice (*P* < 0.001), while it was also differed by age, but it was not statistically significant (*P* = 0.304). Dentists aged ≥ 40 years and those with more than 10 years of experience showed higher proportions of medium and high clinical knowledge. Similarly, dentists working in government hospitals demonstrated comparatively higher clinical knowledge levels than dentists working in private settings. Regarding CME attendance, dentists who never attended CME activities had a slightly higher proportion of medium and high clinical knowledge, but it was not statistically significant.

**Table 4:** Distribution of clinical and risk factor knowledge by characteristics of dentists

	Clinical Knowledge of OC			Risk Factor Knowledge of OC		
	Low *N* (%)	Medium and High *N* (%)	*P* value	Low *N* (%)	Medium and High *N* (%)	*P* value
Sex			0.348			1.000
Male	93 (63.3)	54 (36.7)		48 (32.7)	99 (67.3)	
Female	46 (70.8)	19 (29.2)		21 (32.3)	44 (67.7)	
Age, years			0.304			0.713
23–29	93 (67.4)	45 (32.6)		43 (31.2)	95 (68.8)	
30–39	36 (66.7)	18 (33.3)		20 (37.0)	34 (63.0)	
≥ 40	10 (50.0)	10 (50.0)		6 (30.0)	14 (70.0)	
Years of practical experience			0.015			0.468
< 5 years	83 (73.5)	30 (26.5)		40 (35.4)	73 (64.6)	
5–10 years	44 (60.3)	29 (39.7)		23 (31.5)	50 (68.5)	
> 10 years	12 (46.2)	14 (53.8)		6 (23.1)	20 (76.9)	
Scope of practice			0.452			0.542
General DP	92 (67.6)	44 (32.4)		42 (30.9)	94 (69.1)	
Specialty DP	47 (61.8)	29 (38.2)		27 (35.5)	49 (64.5)	
Place of practice			< 0.001			0.718
Government hospital	16 (34.0)	31 (66.0)		13 (27.7)	34 (72.3)	
Private hospital	18 (75.0)	6 (25.0)		8 (33.3)	16 (66.7)	
Private dental clinic	105 (74.5)	36 (25.5)		48 (34.0)	93 (66.0)	
Attended CME			0.701			0.563
Yes	25 (69.4)	11 (30.6)		10 (27.8)	26 (72.2)	
Never	114 (64.8)	62 (35.2)		59 (33.5)	117 (67.5)	

In contrast, risk factor knowledge did not show significant variation across any of the examined characteristics, including sex, age, years of experience, scope of practice, place of practice, or CME attendance (all *P* > 0.05).

Table 5 shows the multivariate logistic regression analysis of factors associated with oral cancer knowledge. Higher knowledge was significantly associated with years of practical experience (*P* = 0.037), with dentists having 5–10 years (OR = 2.02; 95% CI: 1.03–3.96) and > 10 years (OR = 2.77; 95% CI: 1.07–7.23) of experience showing increased odds compared to those with < 5 years. Place of practice was also significant (*P* < 0.001), with government hospital dentists demonstrating substantially higher odds of greater knowledge (OR = 5.51; 95% CI: 2.65–11.46).

**Table 5:** Results of multivariate logistic regression of oral cancer knowledge

	OR	95% CI	*P* Value
Years of practical experience			0.037
< 5 years	1		
5–10 years	2.02	1.03–3.96	
> 10 years	2.77	1.07–7.23	
Place of practice			< 0.001
Private dental clinic	1		
Private hospital	0.86	0.31–2.40	
Government hospital	5.51	2.65–11.46	

## Discussion

This study examined dentists’ knowledge, attitudes, and clinical practices related to oral cancer within a diverse professional population practicing in Kabul. The findings indicate that while participants demonstrated strong awareness of major behavioral risk factors, their clinical knowledge of oral cancer presentations was comparatively limited.

Clinical knowledge scores were modest, with most dentists falling into the low-knowledge category. Although participants were largely familiar with fundamental oral examination procedures, and correctly identified squamous cell carcinoma as the predominant oral malignancy, notable gaps were observed in their recognition of common anatomical sites and associated clinical features of oral cancer. Dental qualification in Afghanistan, a 6-year Doctor of Dental Medicine (DMD) or Stomatology degree, covers basic sciences, pre-clinical training, and clinical rotations across major dental specialties, concluding with a mandatory internship to ensure both theoretical knowledge and practical skills, including areas like oral cancer detection. The gaps identified in clinical knowledge and diagnostic confidence in the present study suggest that undergraduate exposure alone may not be sufficient to ensure sustained competence in recognizing early oral cancer in routine practice. Similar gaps have been reported in other studies, where dentists showed limited awareness of the anatomical distribution and presentation patterns of oral cancer lesions [14, 15, 17].

By contrast, participants demonstrated relatively strong awareness of most established risk factors, particularly tobacco use, Naswar, alcohol consumption, paan chewing, and a history of oral cancer lesions. These findings are consistent with studies from other countries where dentists typically demonstrate higher knowledge of behavioral and lifestyle-related risks compared with clinical diagnostic features [19, 22–25]. However, awareness of age-related and dietary risk factors was less consistent, mirroring trends reported elsewhere [19, 23, 25, 26].

In this study, dentists generally expressed positive attitudes toward oral cancer screening, early detection, and patient counselling. Most supported annual screening for adults aged 40 years or older, recognized the importance of early detection for improving survival outcomes, and incorporation of tobacco cessation counselling into routine dental practice. However, confidence in performing oral cancer examinations varied, with a substantial proportion of dentists expressing uncertainty regarding their training and clinical skills. This finding is consistent with previous studies documenting limited self-perceived competency among dentists despite positive attitudes [10]. The view that oral cancer examinations should be a reimbursable procedure may suggest a presence of perceived barriers related to time constraints, resource availability, and the need for additional training.

Clinical engagement with diagnostic procedures was relatively low, with most dentists reporting no biopsy or referral of suspicious lesions within the past year. Similar trends have been observed in other studies where dentists relied predominantly on specialist referral instead of performing initial diagnostic interventions themselves [11]. Although specialist referral is an integral component of oral cancer care, reliance on referral alone may introduce delays and additional barriers to care in settings such as Afghanistan, where specialist consultations and diagnostic procedures are largely paid out of pocket. Strengthening dentists’ capacity to perform initial diagnostic interventions at the primary care level may help support timely evaluation and management of suspicious oral lesions, although the effectiveness of such approaches would require further investigation. In addition, while most participants routinely assessed general tobacco and alcohol use during history-taking, fewer inquired about specific consumption patterns, which may limit effective risk stratification and tailored patient counselling.

A notable finding of this study is the limited availability of oral cancer awareness materials in the dental clinics observed, with nearly 70% of dentists reporting that no educational resources were accessible to patients. The limited availability of patient education materials identified by the participants suggests potential gaps in preventive resources within dental clinics. Previous studies suggest that culturally appropriate educational materials may support patient awareness and engagement [27], suggesting a potential area for future development to improve clinical environments.

Clinical knowledge was significantly associated with years of experience and practice setting, with higher knowledge levels observed among more experienced dentists and those working in government hospitals. This pattern is consistent with previous studies linking exposure, caseload, and institutional support to increased diagnostic familiarity [17, 23, 28]. CME attendance was not statistically associated with knowledge levels in this study. This finding may be explained by the limited availability and low uptake of CME programs in Afghanistan, where only 36 (16.98%) dentists reported participation. CME activities have been introduced only recently, are not mandatory, and do not offer incentives such as promotion or academic credits, which are likely to contribute to low engagement. Attendance may also have been limited to a single session and not necessarily focused on oral cancer. In this context, many Afghan dentists may rely primarily on clinical experience and self-directed learning rather than formal continuing education to develop their knowledge. In contrast, studies from other settings have reported that CME attendance and fewer years of professional experience are positively associated with higher knowledge levels [24, 29–31]. Further research is needed to explore the content, accessibility, and potential impact of CME programs in Afghanistan.

### Limitations

Several limitations of this study should be acknowledged. First, due to the cross-sectional design and nature of the study, changes in knowledge, attitudes, or practices over time were not assessed. Second, data was collected using a self-administered questionnaire which may introduce recall bias or social desirability bias, potentially leading to over-reporting of more favorable responses, with overestimation of knowledge and/or positive practices. However, the questionnaires were completed by the participants alone, without the use of an interviewer, which may potentially reduce the influence of social desirability on their responses. Third, the use of convenience sampling and the study population limited to dentists practicing in, Kabul City (capital of Afghanistan), may limit generalizability of the findings to other regions of Afghanistan, particularly rural areas where access to training, patient populations, and clinical resources may differ.

### Implications for practice

The findings highlight several areas that may assist with supporting oral cancer prevention and early detection within dental practices, including: targeted clinical training focusing on oral cancer diagnostic features and lesion recognition; standardization and improvement of CME content to ensure competence-based learning; increased availability of culturally appropriate patient education materials to support patient awareness and earlier self-referral; encouragement of more structured and comprehensive risk assessment protocols to improve documentation and may enhance individualized patient counseling.

## Conclusion

Dentists practicing in Kabul demonstrated strong awareness of major behavioral risk factors for oral cancer but comparatively limited knowledge of its clinical presentation. Although attitudes toward screening were generally positive, gaps in training, clinical confidence, and availability of educational materials were identified. Addressing these gaps through targeted training, improved access to CME programs, and enhanced clinical resources, including patient awareness materials, may help support oral cancer prevention and detection efforts in the region.

## Data Availability

Data is available from the corresponding author upon reasonable request, subject to approval by the Institutional Review Board.
